# Danon disease: Two case reports and literature review

**DOI:** 10.1097/MD.0000000000046993

**Published:** 2026-01-16

**Authors:** Kun Yu, Bingnan Peng, Dandan Zhao, Ying Wang

**Affiliations:** a Department of Neurology, Zibo Central Hospital, Zibo City, Shandong Province, China; b Department of Internal Medicine, Badou Health Center, Zibo City, Shandong Province, China; c Department of Neurology, Shandong Key Laboratory of Mitochondrial Medicine and Rare Diseases, Research Institute of Neuromuscular and Neurodegenerative Diseases, Qilu Hospital of Shandong University, Jinan, Shandong Province, China.

**Keywords:** cardiomyopathy, case report, Danon disease, LAMP2 gene, LAMP2 mutation

## Abstract

**Rationale:**

Danon disease (DD) is an X-linked lysosomal storage disorder caused by *LAMP2* variants, with males presenting more severe phenotypes. However, evidence for genotype-phenotype correlation remains limited. This study reports 2 male DD patients with distinct *LAMP2* mutations to clarify mutation-specific prognostic differences.

**Patient concerns:**

A 15-year-old male: chest tightness and palpitations, creatine kinase (CK) 3867 U/L, and hypertrophic cardiomyopathy. A 12-year-old male: exertional dyspnea and syncope, left ventricular ejection fraction 36%, left ventricular thrombus, and CK 5210 U/L.

**Diagnoses:**

Both were diagnosed with DD via genetic testing: the 15-year-old had a *LAMP2* IVS6 + 1G > T splice mutation, and the 12-year-old carried a *LAMP2* exon 1 deletion.

**Interventions:**

The 15-year-old underwent heart transplantation followed by immunosuppressive therapy. The 12-year-old received only symptomatic treatment without transplantation.

**Outcomes:**

The 15-year-old had normal cardiac function and normalized CK levels during 24-month posttransplant follow-up. The 12-year-old died of heart failure 8 months after diagnosis.

**Lessons:**

*LAMP2* mutation types correlate with DD severity. Heart transplantation improves prognosis in severe cases, emphasizing the importance of early diagnosis and intervention.

## 1. Introduction

Danon disease (DD) is rare disease with X-linked multiple organ involvement caused by mutations in the lysosomal-associated membrane 2 (*LAMP2*) gene.^[1–3]^ Due to its X-linked dominant inheritance pattern, the clinical presentation of DD varies between genders – female patients typically have a later symptom onset and better prognosis than males.^[4,5]^ As previously reported, the heart, skeletal muscles, and nervous system are the main organs affected by DD, though the retina, liver, and lungs may also be involved.^[6–8]^ Cardiac manifestations are usually the most prominent and severe, whereas skeletal muscle and neurological phenotypes vary considerably.^[3]^ For cardiac involvement, hypertrophic cardiomyopathy (HCM) and arrhythmias (especially preexcitation syndrome) are the most common manifestations, and the presence of hypertrophic cardiomyopathy is an indicator of a poor prognosis for the patient.^[9]^ Regarding musculoskeletal symptoms, male patients are more likely to have symptomatic muscle involvement, along with abnormalities in AST, ALT, and LDH (aspartate aminotransferase, AST; alanine transaminase, ALT; lactate dehydrogenase, LDH; all units: U/L).^[2]^ As for the involvement of nervous system, the main manifestation is cognitive impairment.^[3]^

The *LAMP2* gene is localized to the X-chromosome (Xq24) and consists of 9 exons. Exon 9 undergoes alternative splicing, generating 3 spliced isoforms: LAMP-2a, LAMP-2b, and LAMP-2c.^[3]^ These isoforms differ in 3 key aspects: the functions of their transmembrane and cytoplasmic domains, their tissue distribution, and their roles in the autophagy degradation pathway.^[3]^ It is predicted that most *LAMP2* gene mutations lead to the deficiency of all 3 LAMP2 isoforms.^[3]^ As of now, isoform-specific mutations have only been found in LAMP2, suggesting that a deficiency of LAMP-2b alone is enough to trigger DD.^[3]^ The distribution of LAMP2 proteins in the human body exhibits tissue specificity, with LAMP-2a and LAMP-2b being abundantly distributed in tissues such as liver, lung and placenta, while LAMP-2b is predominantly found in the heart, skeletal muscle and brain. The pathogenesis and manifestations of DD are largely attributed to the lack of LAMP-2b. LAMP2 is a 95 to 120 kDa cytoplasmic protein and mainly localizes in lysosomes, endosomes, and autolysosomes.^[1]^ Recent research has revealed that the underlying mechanism involves a disruption in autophagy. This disruption results in the impaired fusion of autophagosomes with lysosomes and/or inefficient biogenesis and maturation of lysosomes.^[3]^

The pathological features of the disease are the presence of cytoplasmic vacuoles and/or granules in muscular tissue, which exhibit increased activity of lysosomal enzymes such as acid phosphatase and nonspecific esterases.^[10]^

With respect to the treatment of DD, currently, heart transplantation is the only radical treatment method for patients with HCM and heart failure. Without heart transplantation, prognosis is poor: male patients rarely survive past 30 years of age, while females rarely survive past 50 years of age.^[3,11]^ In addition, gene editing therapy is also promising for the treatment of DD. In a *LAMP-2b*-deficient mouse model, AAV9-mediated *LAMP-2b* treatment significantly improves muscle strength and effectively reduces the accumulation of abnormal autophagic vesicles inside myocytes.^[12]^ CRISPR-Cas9 combined with single-stranded DNA can correct *LAMP2* mutations in patient, enhancing ATP production, oxygen consumption and maximum respiratory capacity, salvaging some DD phenotypes in vitro.^[13]^

Herein, we report 2 cases of DD (Clinical trial number: KYLL-202204-042-1). In 1 case, there is a novel intronic mutation in the *LAMP2* gene (IVS6 + 1G > T), while in the other case, an exon 1 deletion mutation in *LAMP2* is observed.

## 2. Case presentation

### 2.1. Case 1

**Figure 1.**
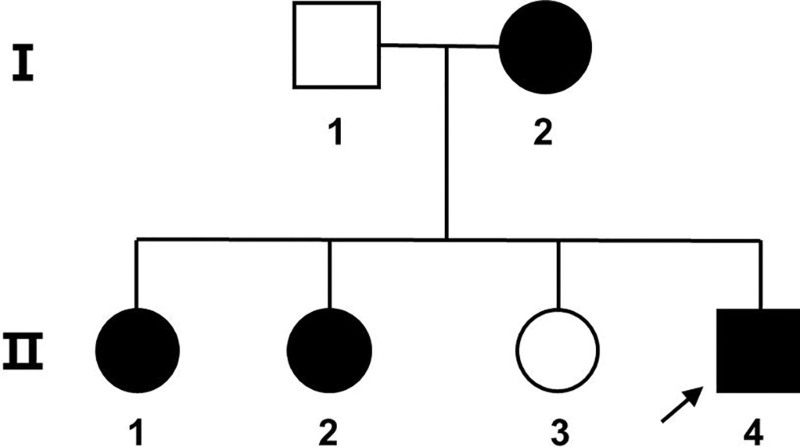
Pedigree of the family with LAMP2 mutation. II-4 is proband. Black symbols indicate affected subjects, white symbols indicate unaffected subjects. The black arrow indicatesthe proband. LAMP2 = lysosomal-associated membrane protein 2.

On May 8, 2008, a 15-year-old male proband (II-4) in early adolescence was admitted to the hospital. He presented with a 3-year history of elevated creatine kinase (CK) and transaminase levels (Fig. 1). In 2005 – three years before this admission, he experienced recurrent episodes of palpitations and was diagnosed with supraventricular tachycardia. Following radiofrequency ablation, the palpitations resolved. However, preoperative examinations revealed that his CK levels were markedly elevated, ranging from 5 to 10 times above normal values. Additionally, his ALT, AST and LDH levels were mildly increased. Furthermore, the patient typically showed no symptoms of skeletal muscle impairment, such as weakness, atrophy, fatigability, exercise intolerance, myalgia, or muscle pain. The patient had been thin since childhood, yet there were no abnormalities in motor development. Besides, due to frequent medical visits, the patient had poor academic performance and dropped out of elementary school.

**Figure 2.**
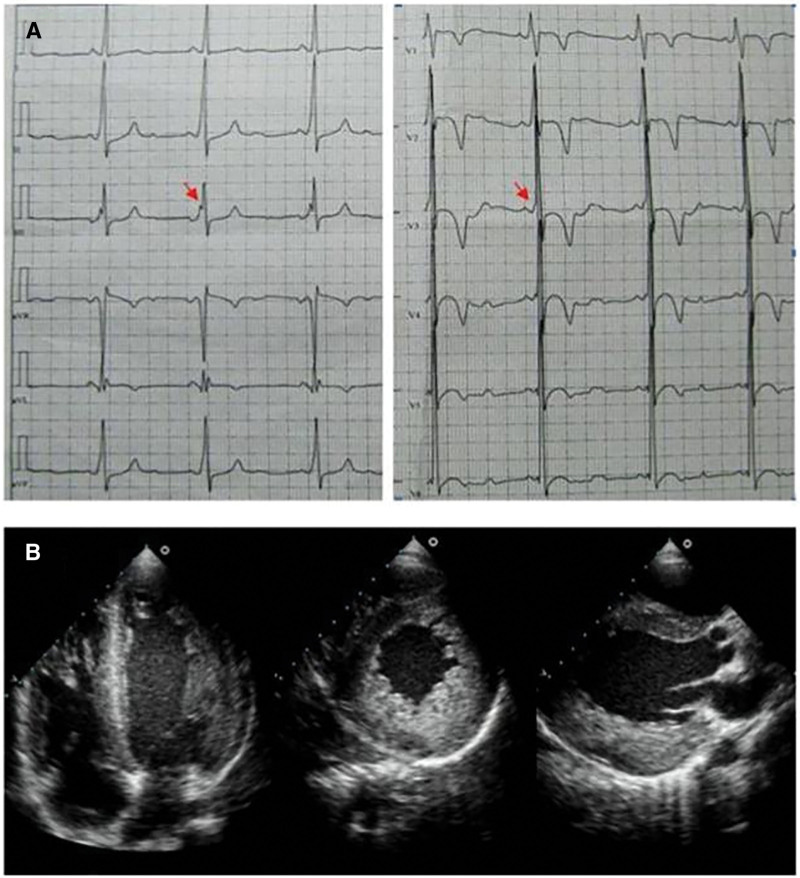
Electrocardiogram and echocardiography of the proband aged 15 years. (A) ECG shows sinus rhythm, premature atrial contractions, WPW syndrome. The typical delta wave on the ECG of WPW syndrome (arrows) (B) Echocardiography assessment shows left ventricular hypertrophy. ECG = electrocardiogram, WPW = Wolff–Parkinson–White.

**Figure 3.**
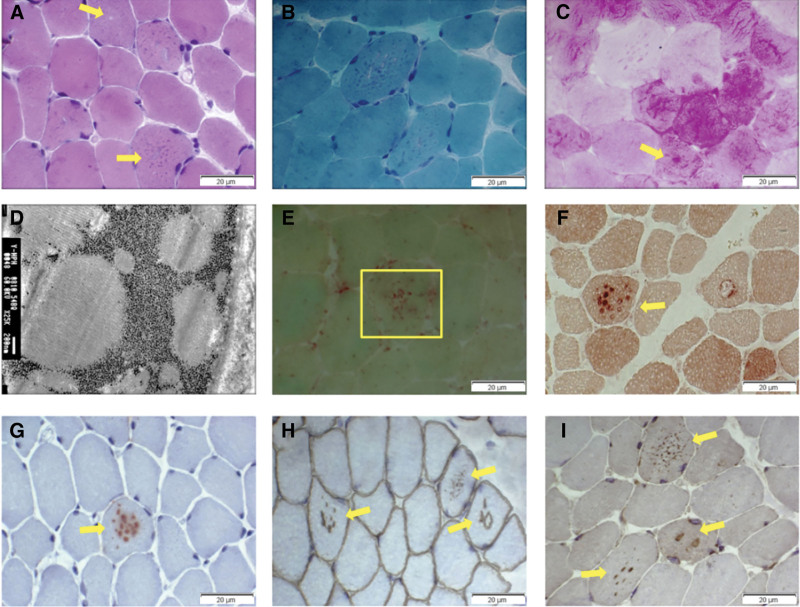
Histological staining of the proband’s skeletal muscle.(A) HE staining showed small vacuoles (arrows) within the muscle fibers containing basophilic particels; (B) Golgi staining showed the absence of RRF and RV in myofibers; (C) PAS staining (d,×400) showed abnormal glycogen granules (arrows) deposition in muscle fiber vacuoles; (D) electron microscopy revealed many glycogen particles deposited in the cytoplasm; (E–G) The staining of ACP, NSE and AchE in vacuoles showed positive results (arrows). (H) Immunohistochemical analysis demonstrated high expression of dystrophin both on myofiber membrane and in vacuoles (arrows); (I) Immunohistochemistry demonstrated only a few intracytoplasmic vacuoles displayed the expression of LAMP2 protein (arrows). AchE = acetylcholinesterase, ACP = acid phosphatase, HE = hematoxylin-eosin, LAMP2 = lysosomal-associated membrane protein 2, NSE = nonspecific esterase, PAS = periodic acid-Schiff staining, RRF = ragged-red fibers, RV = irregular vessels.

On the day of admission, the physical examination showed no significant abnormalities, with the exception of emaciation. Biochemical examination on admission indicated: CK, 1555 U/L (reference range: 24–195 U/L); CK-MB, 48.1 ng/mL (reference range: 0–15 ng/mL). Electrocardiogram (ECG) showed Wolff–Parkinson–White (WPW) syndrome, left ventricular hypertrophy, ST-T abnormalities (Fig. 2A). ECG revealed concentric left ventricular hypertrophy: interventricular septum thickness diastolic (IVSTd), 17 mm; left ventricular end diastolic diameter (LVDd), 27 mm; left ventricular ejection fraction (LVEF), 60% (Fig. 1B). No characteristic changes were observed in electromyography (EMG). In combination with the symptoms, physical examination findings, laboratory tests results, and medical history of the patient, we suspected DD as the underlying cause. To confirm this suspicion, a biopsy of the left biceps muscle was conducted. HE (hematoxylin-eosin) staining showed that the muscle fibers had small vacuoles, with basophilic particles present inside these vacuoles (Fig. 3A). Golgi staining showed the absence of ragged-red fibers and irregular vessels in myofibers (Fig. 3B), the periodic acid-Schiff staining (PAS) staining showed abnormal glycogen granules deposition in muscle fiber vacuoles (Fig. 3C), electron microscopy showed many glycogen particles deposited in the cytoplasm (Fig. 3D), and the staining of acid phosphatase (ACP), nonspecific esterase (NSE) and acetylcholinesterase (AchE) in vacuoles showed positive results (Figs. 3E–G). Anti-dystrophin staining showed expression of dystrophin protein in intracytoplasmic vacuoles of diseased muscle fibers. This indicated that autophagic vacuoles have sarcolemmal features (Fig. 3H). Furthermore, immunohistochemical analyses using a commercial antihuman LAMP2 antibody demonstrated only a few intracytoplasmic vacuoles displaying the expression of LAMP2 protein (Fig. 3I).

**Figure 4.**
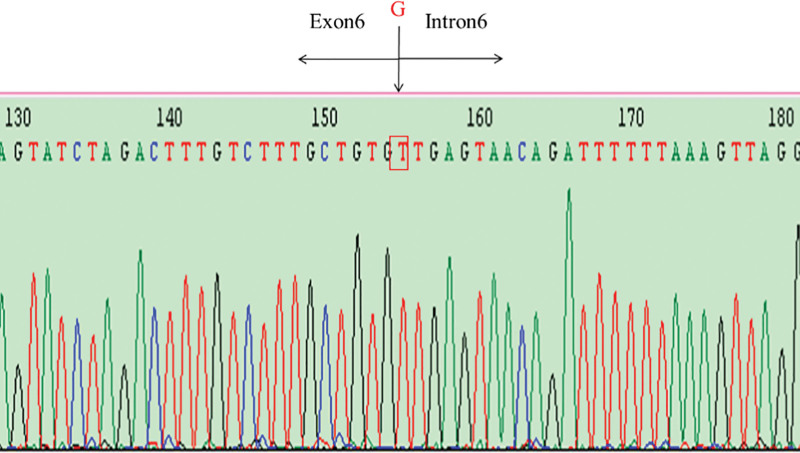
The schematic diagram of LAMP2 gene mutation shows that the first G of the intron 6 is replaced by T. LAMP2 = lysosomal-associated membrane protein 2.

In terms of family’s genetic history, his mother (I-2) (Fig. 1) was diagnosed with ‘hypothyroidism’ at the age of 32. At the time of the patient’s admission, she was 42, with normal CK, AST: 46 to 59 U/L, LDH: 319 to 448 U/L (reference range: 114–240 U/L); ECG showed atrial fibrillation and ST-T changes; and echocardiogram showed no abnormalities. Additionally, all 3 of his siblings had normal myocardial enzyme levels, electrocardiogram results, and echocardiogram finding. To identify the genetic basis underlying the proband’s condition and the family’s clinical phenotypes, genomic DNA was extracted from the peripheral blood of the proband, his mother and his siblings. Specifically, genetic testing was performed using Sanger sequencing to analyze the entire coding region (exons 1–9) and flanking intronic sequences of the *LAMP2* gene. For standardized variant classification, the 2015 American College of Medical Genetics and Genomics (ACMG) guidelines were followed. To verify whether the identified variant was inherited within the family, Sanger sequencing was also used to verify whether this variant was present in other family members. Ultimately, a novel splicing mutation referred to hemizygous mutation (IVS6 + 1G > T) in intron 6 of *LAMP2* (Fig. 4) was identified. It was predicted to cause abnormalities in structural and functional domains of the protein. Upon analyzing the family segregation of the mutation, the same mutation was also found in his mother, sisters (II-1, II-2), whose electrocardiogram and echocardiography were normal, whereas the mutation was not detected in his third sister (II-3) (Fig. 1). Fortunately, the patient underwent a heart transplant in 2020 and has lived to the present.

### 2.2. Case 2

**Figure 5.**
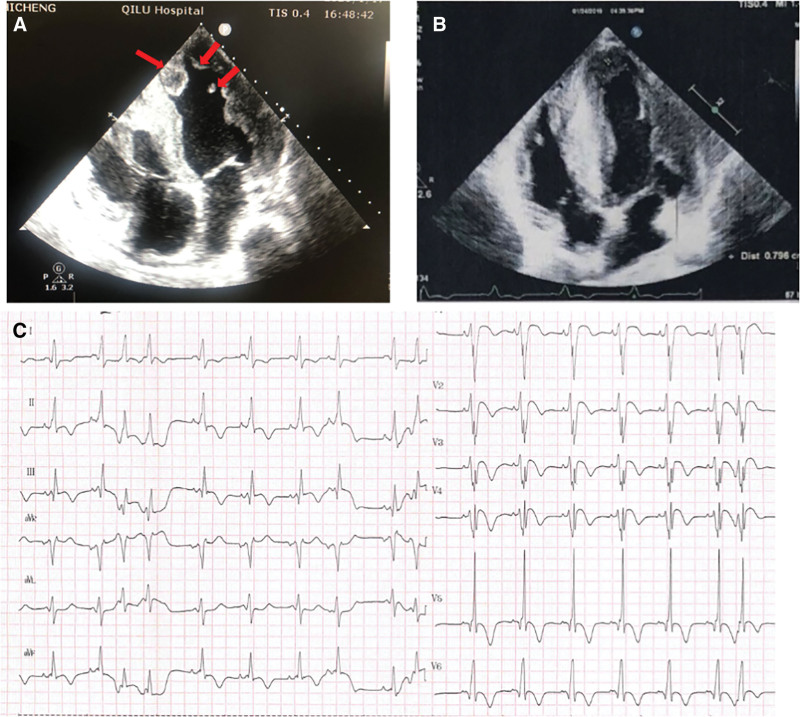
The echocardiogram and ECG of the patient. (A) The patient’s first echocardiogram and (B) the rechecked echocardiogram after the patient took the anticoagulant warfarin. The thrombus is pointed to by the red arrow. (C) The patient’s ECG indicates atrial premature beats. ECG = electrocardiogram.

In January 2019, a 12-year-old boy was admitted to the hospital due to fever, cough, dizziness, and chest tightness, and his echocardiography revealed cardiac hypertrophy, left ventricular thrombus, and LVEF of 36% (Fig. 5A). He was given warfarin anticoagulation and ceftriaxone anti-infective treatment, and then transferred to Qilu Hospital of Shandong University for further treatment. Earlier in his life, at 1 year of age, the patient was diagnosed with hypertrophic cardiomyopathy, which was untreated. The boy showed signs of weakness from childhood: his ability to tolerate exercise was worse than that of his peers, and he would easily become tired or short of breath following activity. There were no abnormalities noted in the patient’s intelligence. His mother had been diagnosed with renal insufficiency in the past, while his father and sister had no known health issues.

**Figure 6.**
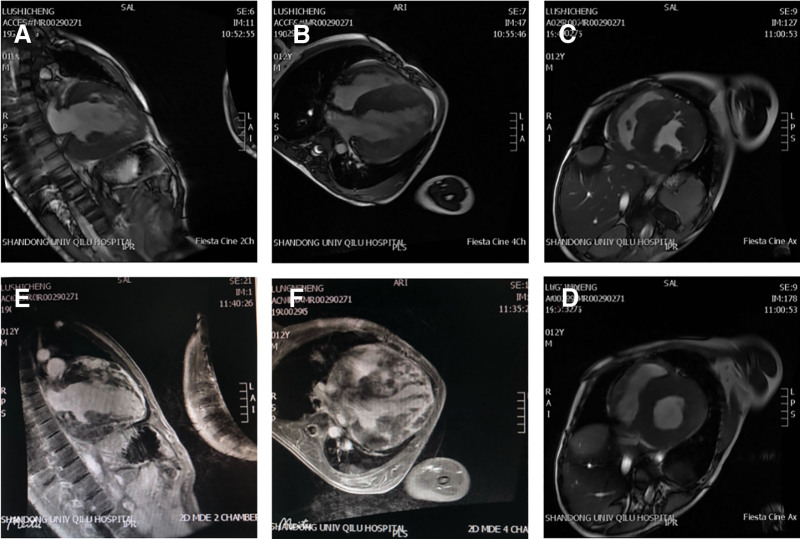
Cardiac magnetic resonance imaging (CMR) of the patient. (A, B) Diffuse thickening of the left and right ventricular myocardium; (C, D) MR first-pass myocardial perfusion imaging showing multiple areas of hypoperfusion in the left ventricular myocardium, suggestive of myocardial ischemia; (E, F) MR delayed-enhancement scanning revealing multiple foci of abnormal enhancement in the left and right ventricular myocardium, indicative of scarring or fibrosis.

**Figure 7.**
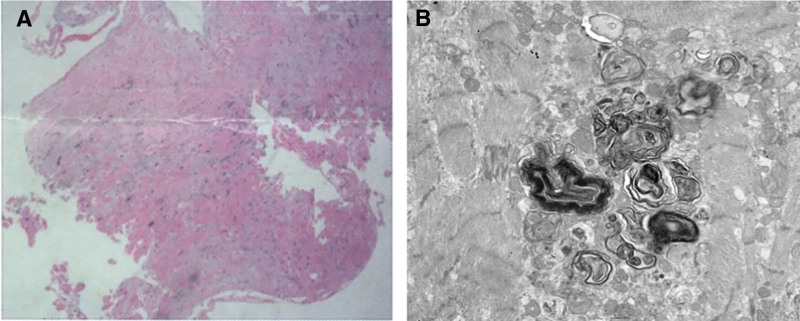
Myocardial histological staining of the patient. (A) HE staining and (B) electron microscopy of cardiomyocyte. HE = hematoxylin-eosin.

**Figure 8.**
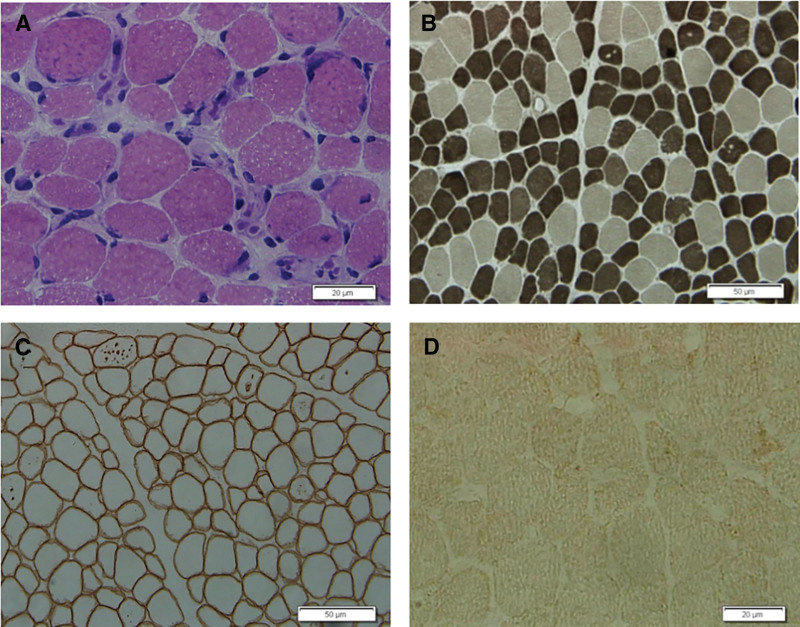
Histological staining of the patient’s skeletal muscle.(A) HE staining showed many small vacuoles and containing basophilic particels within the muscle fibers; (B) ATP staining demonstrated selective atrophy of type Ⅱ muscle fibers (pH = 10.6); (C) Immunohistochemical analysis using an antibody against dystrophin revealed the presence of dystrophin protein on the membrane of muscle fibers and vacuoles; (D) Immunohistochemistry of skeletal muscle biopsy showed a near absence of LAMP2 protein in muscle fibers. HE = hematoxylin-eosin, LAMP2 = lysosomal-associated membrane protein 2.

On admission, neurological examination revealed gross nystagmus in both eyes. Laboratory findings showed markedly elevated CK, LDH, AST and ALT: ALT 136 U/L (reference range: 9–50U/L), AST 159 U/L (reference range: 15–40U/L), Urea Nitrogen 9.40↑mmol/L (reference range: 2.30–7.80mmol/L), CK 482 U/L (reference range: 38–174U/L), CK-MB 9.5 ng/mL (reference range: 0.3–4.0ng/mL), LDH 980 U/L (reference range: 120–230U/L), international normalized ratio 1.37 (reference range: 0.8–1.2), prothrombin time (PT-S) 15.2 seconds (reference range: 8.8–13.8s), erythrocyte sedimentation rate: 48 mm/h (reference range: 0–15 mm/h). Amino-terminal pro-B-type natriuretic peptide (NT-proBNP) level was 10,200 pg/mL (reference range: <300 pg/mL). ECG showed frequent atrial and ventricular premature beats (Fig. 5C). A repeat echocardiogram was performed later, which showed that the left ventricular thrombus had disappeared, and additional parameters were noted: IVSTd, 34 mm; left atrial dimension, 35mm; LVDd, 39 mm; pulmonary artery systolic pressure, 28 mm Hg; LVEF, 40% (Fig. 5B). Cardiac magnetic resonance showed diffuse thickening of the left and right ventricular myocardium (Fig. 6A and B), MR first-pass myocardial perfusion showed multiple areas of hypoperfusion in the left ventricular myocardium suggestive of myocardial ischemia (Fig. 6C and D), and MR delayed-enhancement scanning showed multiple foci of abnormal enhancement in the left and right ventricular myocardium suggestive of scarring or fibrosis (Fig. 6E and F). Histological analysis of cardiac muscles reveals disturbed myofibrillar disruption and atrophy, hypertrophy and lysis necrosis of some cardiomyocytes, and proliferation of fibrous tissue (Fig. 7A). Electron microscopic examination of the cardiac tissue showed myelin-like vesicles in cardiomyocytes. Skeletal muscles also showed scattered small basophilic granules in myofibers (Fig. 7B). To further confirm the diagnosis, the patient also underwent a skeletal muscle biopsy. HE staining of the skeletal muscle biopsy sample revealed numerous small vacuoles along with basophilic particles within the muscle fibers (Fig. 8A). ATP staining (pH = 10.6) demonstrated selective atrophy of type Ⅱ muscle fibers (Fig. 8B). Immunohistochemical analysis, employing an antibody against dystrophin and LAMP2, showed the presence of dystrophin protein on both the membrane of muscle fibers and within the vacuoles (Fig. 8C), while an almost complete absence of LAMP2 protein in muscle fibers (Fig. 8D). Given that the skeletal muscle histological results were consistent with vacuolar myopathy, and combined with immunohistochemistry findings that suggested a high likelihood of Danon disease (DD), the clinical team suspected the patient had DD. To confirm the suspected diagnosis, further next-generation sequencing was performed – and it demonstrated a deletion variant in exon 1 of the *LAMP2* gene. Next-generation sequencing was conducted on an Illumina MiSeq platform with a targeted panel covering the *LAMP2* gene. Variant classification followed the 2015 ACMG guidelines. Family members were also tested with the same panel to confirm segregation of the *LAMP2* deletion. This deletion was confirmed as a hemizygous loss spanning the entire exon 1 (reference transcript: NM_002294.3; specific base pair coordinates unavailable due to long-term data loss and family inability to provide supplementary records) via quantitative PCR (qPCR). Family segregation analysis confirmed it was a de novo variant, as no such deletion was detected in the patient’s parents or sister. The patient was admitted to Qilu Hospital of Shandong University on 2 occasions due to heart failure and recurrent infections. Regrettably, in May 2020, following the exacerbation of his condition, the patient passed away at a local hospital.

## 3. Discussion

This study presents 2 detailed cases of male patients diagnosed with Danon disease (DD), a rare X-linked lysosomal storage disorder driven by *LAMP2* mutations. By systematically documenting their clinical trajectories, we contribute critical new insights to the understanding of pediatric DD. Case 1 harbored a novel hemizygous splicing mutation (IVS6 + 1G > T) in intron 6, while Case 2 presented a de novo hemizygous deletion spanning the entire exon 1 of *LAMP2*. These distinct genetic variants, paired with contrasting clinical outcomes, make the cases valuable for expanding DD’s clinical and genetic database.

When comparing the 2 cases, we found clear differences. Case 1 exhibited relatively mild cardiac involvement: elevated CK, WPW syndrome, and hypertrophic cardiomyopathy (HCM), but maintained a LVEF of 60%. In contrast, Case 2 (12-year-old) presented with severe cardiac impairment: LVEF as low as 36%, left ventricular thrombus, and subsequent biventricular myocardial fibrosis. This discrepancy directly translated to outcomes. Case 1 underwent successful heart transplantation and remains alive to date, whereas Case 2’s rapid disease progression prevented transplantation, leading to death before adulthood.

Fundamentally, Danon disease (DD) is a rare X-chromosome dominant disorder caused by mutations in the gene encoding *LAMP2,* leading to reduced or absent *LAMP2* function, causing multiple target organ pathologies, particularly cardiac and skeletal muscle cell autophagy and glycogen deposition and vacuole formation. Epidemiological data indicate that DD is more prevalent among males than females. Compared with female patients, male patients typically experience an earlier onset of the disease, with more severe symptoms, faster disease progression, and a worse prognosis. Clinically, DD is characterized by a classic triad: significant cardiac hypertrophy, skeletal muscle weakness with atrophy, and intellectual disability, which may be also accompanied by electrocardiographic changes in left ventricular preexcitation. Almost all male patients have cardiomyopathy and high CK level.^[3]^ Consistent with this epidemiological feature, both patients we presented are male, with disease onset in childhood. They both have cardiac hypertrophy. However, neither shows obvious cognitive impairment or skeletal muscle lesion symptoms.

To date, more than 110 mutations in the *LAMP2* gene have been identified, and the type of mutation correlates with the severity of the phenotype, with mutations in exons 1 to 8 being clinically severely manifested.^[14]^ Previously reported *LAMP2* intronic mutations cause DD via disrupted pre-mRNA splicing, and the resulting phenotypes depend on the level of residual LAMP2 protein function. Intron 1 mutation leads to mild adult-onset hypertrophic cardiomyopathy (HCM), mild CK elevation, and good prognosis. Intron 5 mutation results in adolescent-onset HCM with WPW syndrome and moderate CK elevation. Intron 6 is a well-documented mutation hotspot in the LAMP2 gene: IVS6 + 5G > C causes HCM with paroxysmal supraventricular tachycardia, while IVS6 + 1G > T (Case 1) induces similar HCM/WPW but allows partial normal splicing, enabling posttransplant survival. Intron 7 mutation (c.560-1G > A) leads to early childhood-onset severe HCM, left ventricular dysfunction, and need for early transplantation. Overall, intronic mutation severity correlates with residual LAMP2 expression.

The patient in Case 2 has a deletion in exon 1 of the *LAMP2* gene, with early onset and severe symptoms. In Case 1, by contrast, it is a family pedigree with the IVS6 + 1G > T mutation in the *LAMP2* gene. The proband in Case 1 has relatively mild symptoms, mainly characterized by an increase in CK and WPW syndrome. The muscle pathology results suggest the deposition of small basophilic vacuoles and basophilic granular substances within the muscle fibers. Considering the symptoms of the proband and the results of the genetic sequencing, this proband is diagnosed with DD. However, the immunohistochemical result of the muscle biopsy of the proband shows that the LAMP2 protein is positive, which may be related to the fact that it is a point mutation in the noncoding region of intron 6, which does not completely block protein synthesis. Muscle pathology shows *LAMP2* nonsense, splice, or exon 9b mutations lead to either trace protein or no significant level drop.^[15]^ Additionally, in female patients with DD, the detection of LAMP2 protein reveals a mosaic distribution of LAMP2 positive and negative muscle fibers.^[16]^ Mosaic LAMP2 expression in female DD patients explains their milder, later-onset phenotypes and is diagnostically critical: it distinguishes asymptomatic carriers from noncarriers and avoids misdiagnosis with myopathies showing uniform LAMP2 expression, aiding early surveillance.

It is worth noting that IVS6 + 1G > T is a novel mutation in the noncoding region of the *LAMP2* gene, which have not been reported. Previous case reports have shown that the IVS6 + 5G > C mutation of the *LAMP2* gene can lead to the skipping of exon 6 of the posttranscriptional mRNA, and point mutations in introns 1 and 5 can result in abnormal splicing of the mRNA.^[17]^ Moreover, in spite of the same *LAMP2* mutation, the affected individuals exhibited varying degrees of clinical features, including hypertrophic cardiomyopathy, skeletal myopathy and developmental delay. The ECG abnormality of the proband in Case 1 first presented as supraventricular tachycardia. After undergoing radiofrequency ablation treatment, the condition improved. However, over a period of 3 years, it gradually progressed to WPW syndrome and hypertrophic cardiomyopathy. Previous case reports suggest that cardiac ablation has not always been successful in DD patients,^[10,18]^ possibly due to the diffuse fibrosis that is resistant to treatment.^[3]^ In Case 2, a limitation exists in that the patient’s *LAMP2* gene can be clearly defined as a deletion in exon 1, but the information about the specific missing base has been lost for too long and the patient’s family was unable to provide it.

Upon comparing these 2 cases, we can draw the conclusion that the symptoms associated with gene fragment deletions tend to be more severe than those resulting from point mutations. Case 1’s IVS6 + 1G > T splice-site mutation likely preserved partial normal splicing and some functional LAMP2, whereas Case 2’s exon 1 deletion completely abolished LAMP2 expression. This functional difference aligned with their clinical divergence: Case 2 had earlier onset, more severe cardiomyopathy, multisystem involvement, and fatal outcome without transplantation, while Case 1 survived post-heart transplantation with milder symptoms – reflecting the impact of partial versus complete LAMP2 deficit.

Given DD’s X-linked dominant inheritance, targeted genetic counseling and familial screening are essential for disease management. For families with confirmed *LAMP2* mutations, asymptomatic female relatives should undergo *LAMP2* carrier screening. Additionally, they need long-term surveillance (annual ECG, echocardiography, CK testing) due to potential late-onset cardiac manifestations. For de novo mutations, parents or siblings typically test negative, but counseling should cover low recurrence risk and offer prenatal testing if desired. This proactive approach aids early identification of at-risk individuals and timely interventions.

Regarding the treatment of cardiomyopathy in DD patient, previous studies have shown that heart transplantation is the only option. However, a recently published Phase 1 clinical trial offers promising insights into the treatment of DD. A single intravenous infusion of RP-A501–a recombinant AAV9 vector carrying the full-length *LAMP-2b* transgene – can improve outcomes in male DD patients who have not undergone heart transplantation. This treatment can increase patient survival, enhance LAMP2 expression in the myocardium, reduce levels of cardiac troponin I and natriuretic peptides, and improve New York Heart Association class and Kansas City Cardiomyopathy Questionnaire-12 (KCCQ-12) scores.^[19]^ Notably, a single infusion of RP-A501 appeared to be generally safe.^[19]^ This represents a significant advancement for patients with DD and provides a new strategy for the treatment of DD. Based on these findings, a global Phase 2 study (NCT06092034) is underway to evaluate the safety and efficacy of RP–A501 (6.7 × 10¹³ genome copies/kg) in male DD patients aged 8 years or older who are afflicted with DD.

Future research in Danon disease (DD) management should target unmet needs. A key priority is advancing AAV9-mediated LAMP2 gene therapy: preclinical models have restored lysosomal function in cardiac and skeletal muscles, but challenges like vector optimization, dosage refinement and long-term safety assessment remain for clinical translation. Another urgent task is expanding *LAMP2* mutation screening in male patients with unexplained cardiomyopathy – particularly those with hypertrophic cardiomyopathy (HCM) and elevated CK. DD’s phenotypic heterogeneity often causes misdiagnosis, and early identification supports timely interventions such as heart transplant referral and targeted family counseling.

In general, the definitive diagnosis of DD mainly relies on gene sequencing. Different gene mutations are associated with distinct symptoms. Even the same point mutation may present different symptoms in various patients. Gene modification therapy may be a new treatment option, but it still requires evaluation through large-scale clinical samples.

## Author contributions

**Data curation:** Dandan Zhao.

**Funding acquisition:** Kun Yu, Ying Wang.

**Investigation:** Bingnan Peng.

**Writing – original draft:** Kun Yu.

**Writing – review & editing:** Ying Wang.
